# Associations between birth order with mental wellbeing and psychological distress in midlife: Findings from the 1970 British Cohort Study (BCS70)

**DOI:** 10.1371/journal.pone.0222184

**Published:** 2019-09-17

**Authors:** Sebastian Stannard, Ann Berrington, Nisreen Alwan

**Affiliations:** 1 Department of Social Statistics and Demography, University of Southampton, Southampton, United Kingdom; 2 ESRC Centre for Population Change, University of Southampton, Southampton, United Kingdom; 3 School of Primary Care, Population Sciences and Medical Education, Faculty of Medicine, University of Southampton, Southampton, United Kingdom; 4 NIHR Southampton Biomedical Research Centre, University of Southampton and University Hospital Southampton NHS Foundation Trust, Southampton, United Kingdom; London School of Hygiene and Tropical Medicine, UNITED KINGDOM

## Abstract

**Background:**

Previous research indicated that birth order was associated with physical health outcomes in adulthood. However, evidence on its association with mental health was lacking. The aim of this study was to investigate if birth order was associated with mental wellbeing and psychological distress at mid-life, stratified by gender, and taking into account confounding factors in childhood and adulthood.

**Method:**

The sample consisted of 9,354 participants of the 1970 British Cohort Study (BCS70). The Warwick Edinburgh Mental Wellbeing Scale (WEMWBS), the Malaise Index and attending a doctor’s consultation in the past year for a mental health issue at age forty-two were used to assess mental wellbeing and psychological distress in midlife. Birth order was ascertained via a parental questionnaire and referred to the numerical birth position of the participants. The associations between birth order, mental wellbeing and psychological distress were tested using linear and logistic regression adjusting for birth characteristics: smoking during pregnancy, maternal age, mother’s marital status, father’s employment, region of birth, parental years of education and parental social class, and factors at age 42: years of education, employment status and partnership status. Potential mediating variables including breastfeeding and birthweight at birth and parental separation and conduct disorder measured at age ten were also taken into account.

**Results:**

We find no evidence to support an association between birth order and midlife psychological distress or attending a doctor’s consultation in both men and women. In unadjusted analysis, there was an association between birth order four and above and a reduced WEMWBS score of -0.79 (95% CI -1.57, -0.02) in men only. This association was attenuated after adjusting for birth characteristics and mediators at birth (0.86, 95% -1.78, 0.07) but was maintained once conduct disorder at age 10 was accounted for (-1.19, 95% CI -2.28, -0.09). However, this association was attenuated once again after adjusting for employment status, years in education and partnership status in adulthood (-1.04, 95% CI -2.11, 0.03).

**Conclusions:**

In this study, birth order was not associated with psychological distress or having a mental health issue at midlife. Accounting for employment status, years of education and partnership status in adulthood attenuated the relationship between birth order and mental wellbeing.

## Background

The WHO defines mental health as a ‘state of wellbeing in which every individual realises his or her own potential, can cope with the normal stresses of life and can work productively and fruitfully’ [1]. High levels of mental wellbeing are shown to reduce the risk of developing chronic diseases and increasing longevity [2–4]. Wellbeing has been identified as a component in reducing mortality in both healthy and unhealthy populations [5], whilst improving survival rates in older generations [6]. Given the ageing population of the UK, it has become increasingly necessary to understand the determinants of positive mental wellbeing and psychological distress into midlife and beyond.

Various psychosocial and socioeconomic circumstances through an individual’s lifecourse, are associated with mental wellbeing and psychological distress in adulthood [2, 3]. However, early life factors such as a person’s birth order, and the impact this may have on mental wellbeing and psychological distress at midlife is less understood. This is despite a growing body of lifecourse literature examining the impact of early life exposures on adult mental health [2, 7].

Theoretical hypotheses regarding the role of birth order have emphasised either its impact on biological conditions within the womb, or the social and environmental conditions a child is raised in. Theoretically, social and biological theories lead to contradictory expectations regarding the association between birth order and adult outcomes. Biological theories emphasise the biological advantage of *not* being the first-born child, whilst sociological theories suggest being the first-born child can have sociological and developmental advantages. The developmental origins of health and disease hypothesis emphases the influence of the fetal environment during pregnancy. The fetus learns to adapt to the environment it expects to enter once outside of the womb, by first adapting to the environment within the womb [8, 9]. Children of different birth orders, in particular first-borns compared to subsequent birth orders, are exposed to changed intra-utero environments and as such will experience different health outcomes later in life. Different utero environments permanently change the body’s structure, physiology, and metabolism [10]. The principle point is that the nutritional, hormonal, and metabolic environment afforded by the mother, will permanently ‘program’ the structure and physiology of her offspring [10].

A first-born child is likely to grow up in a more restricted uterine environment compared to subsequent pregnancies [11]. For example, maternal parity is a well-recognised predictor of birthweight, with the lowest birthweights observed amongst nulliparous women [12]. Low birthweight is associated with an increased risk of schizophrenia [13–16] and a psychiatric diagnosis in adulthood [16]. We hypothesise that birth order may therefore be associated with mental health outcomes at midlife, mediated by birthweight.

Sociological theories focus on resource depletion as a pathway through which children of lower birth orders may receive increased resources and investment compared to subsequent siblings. The eldest sibling represents a better Darwinian bet for survival and reproduction and as such, parents bias investment and resources, both consciously and unconsciously towards the eldest child [17]. Further, as sibship size increases, the resources available for subsequent siblings are depleted [18, 19]. This depletion includes both biological maternal resources such as nutrition, preventative care, and parenting resources, including parental interaction and supervision [19].

To our knowledge there are no existing studies that have investigated birth order, mental wellbeing and psychological distress in midlife. Within a developed country context, birth order has been extensively studied with regards to the development of schizophrenia and has been found to be an independent risk factor for the illness [20–22]. The risk of the illness was generally elevated amongst first-born men (OR 1.5; 95% CI 1.0–2.2) and last-born women (OR 1.3; 95% CI 1.0–1.9)[13, 15, 16, 23]. Other studies, predominantly from the USA, have also found associations between birth order and an increasing linear effect on autism that peaks at birth order two (P<0.01) [24], but no effect of birth order on anorexia was found [25]. Further studies have also linked birth order with accidents. In the UK birth order three or higher increased the risk of experiencing accidents that resulted in hospitalisation (P<0.01) [19]. Birth order has also been connected to birthweight—first order births were smaller than later births for men (3338g vs 3491g, P<0.01) and women (3243g vs 3359g, P <0.01) [26], and infant mortality—in the US later-born infants are more likely to survive (P<0.01) [19]. Whilst multiple sclerosis [27] and irritable bowel syndrome [28] both show a curvilinear relationship with birth order, with first and last order births most likely to develop either disease. Finally, in Sweden, birth orders above two increased the risk of mortality in adulthood (P<0.05) [29].

Two studies using the 1970 British Cohort Study (BCS70) found contrasting evidence. One found an association between exposures in fetal life including low birthweight (P <0.05), early gestational age (P<0.05) and low weight gain (P <0.05) and psychological distress at age twenty-six [30]. However, another study provided weak evidence of an association between birth characteristics including birthweight (men -0.01; 95% CI -0.20–0.18, women -0.01; 95% CI -0.27–0.04), maternal smoking (men 0.07; 95% CI -0.02–0.17, women 0.16; 95% CI 0.07–0.23), breastfeeding (men -0.09; 95% CI -0.18–0.00, women -0.06; 95% CI -0.14–0.01) and adult psychological distress in either the BCS70 or the National Child Development Study (NCDS) [7]. Whilst, other studies have found no evidence of an association between birth order and alcoholism [31], intelligence or the development of personality types [32–34].

Previous studies have also found differences in association when stratified by gender. [20–22]. The risk of schizophrenia was elevated amongst first-born men (OR 1.5; 95% CI 1.0 -2-2) and last-born women (OR 1.3; 95% CI 1.0–1.9) [23]. Men had a linear increasing birth order effect on autism, whereas women represented a V-shaped birth order effect [24]. Psychological illness have also been found to be more common amongst women (mean 2.99) than men (mean 2.16). [30]. These differences in findings were ascribed to parental preference for firstborn boys [17, 18, 31], resulting in parental investment both consciously and unconsciously being distributed unequally across genders [14, 15]. A further study discovered a gender-based difference in parental treatment for the second born child, with a preference for boys [35]. Gender is therefore hypothesised to be an effect modifier resulting in differences in outcomes for men and women, stemming from the unequal distribution of parental resources and investment.

We examined the association between birth order and three mental health outcomes: midlife (age 42 years) mental wellbeing, adult psychological distress and attending a doctor’s consultation, accounting for potential mediation by factors at birth, in childhood and confounding by birth characteristics and factors at age forty-two.

## Method

### Data

The 1970 British Cohort Study (BCS70) has followed 17,096 participants across England, Scotland and Wales born in a single week of 1970, collating information at nine different time points across the life course. A user guide for the dataset can be found at the Centre for Longitudinal Studies [36]. The cohort has also been profiled up to age 34 [37]. This study used a range of survey methods to collect information on the participant’s health, cognitive and social development, education, employment, attitudes and home lives. Information was collected from parents at birth and during childhood, whilst the respondent themselves started to provide information in later childhood. This study used data from the birth and age ten and age forty-two sweep. Ethical approval was granted by the National Health Service (NHS) Research Ethics Committee, and all participants provided fully informed consent. Ethics approval for this analysis was also granted by the University of Southampton Ethics Committee (Reference number: 41778).

### Sample

Within the BCS70 there were 8,578 respondents with valid data on the Malaise Index at and 8,070 with valid data on the WEMWBS at forty-two. This represents 50.5% and 47.49% of the original birth cohort for the Malaise Index and WEMWBS respectively. Previous research using weights to adjust for lost cases, multiple imputation and attrition within the BCS70 were found not to improve the efficiency of models and had weak predicative powers [7, 38]. Weights were therefore not used within this study. We conducted a drop out analysis comparing the sample characteristics at birth and at age ten for all respondents at birth and at age 10 and the respondents followed up at age 42; the output is included in S1 and S2 Tables. Overall, attrition amongst BCS70 cohort member was greatest amongst those with unemployed fathers or absent fathers at birth, who were of low birthweight, who were exposed to maternal smoking, whose parents were single at birth, who were of birth order four and above, and those whose parents were unskilled or partly skilled. Participants who dropped out the sample were therefore more likely to come from disadvantaged backgrounds, linked to class and the presence of an absent father.

### Outcome assessment

Mental health was measured using three instruments; the Malaise Index measuring psychological distress, the Warwick-Edinburgh Mental Wellbeing Scale (WEMWBS) measuring mental wellbeing, and a direct question asking participants whether they had attended a doctor’s consultation in the past year for a mental health issue.

The WEMWBS was designed to capture the presence of positive mental states, with all questions being presented in a positive manner. The scale is comprised of fourteen-questions with five response likert-scale for each question. The responses to each question were summed to produce a single score between fourteen and seventy. A higher WEMWBS score reflects higher levels of positive mental well-being. The scale also indicates a high level of reliability (Cronbach alpha = 0.92) and displays content and criterion validity and acceptable test-retest reliability [39]. The measure was treated as a continuous variable, and unlike the Malaise Index has no defined cut-off to indicate poor mental wellbeing.

The Malaise Index is based on the Malaise Inventory [40]; an established scale to measure signs of psychological distress in adulthood. The index encompasses a set of self-completed questions, combined to produce a measure of psychological distress. The full inventory comprises twenty-four ‘yes-no’ questions; however, the BCS70 has adopted a shortened nine question index. Questions are asked within a standard format, in which one point is awarded for every ‘yes’ response and zero points for every ‘no’ response. When the shortened index is utilised a score of four or higher is an indication of psychological distress [41]. The measure was treated as a categorical variable with a score between zero and three deemed to be at low risk of psychological distress and a score of four or higher deemed to be at high risk [34].

Within the final measure participants were asked to state if they had been to see a doctor or a specialist for a mental health issue in the past year from the time they completed the survey. The measure was treated as a binary outcome with responded either answering ‘yes’ or ‘no’. The decision to analyse this alternative outcome were to test the limitations of the self-reported measures of mental wellbeing and psychological distress, replacing them with a more objective measure of mental health.

### Exposure assessment

Birth order within this study refers to the numerical position a child was born into the family. The measure was derived by subtracting the total number of miscarriages and stillbirths from the total number of pregnancies, to produce the number of live births prior to the participant’s birth. This information was self-reported, collected at the time of the participants birth.

### Covariate assessment

Several childhood factors previously found to be associated with mental wellbeing and psychological distress in adulthood were analysed as potential mediators. A self-reported measure of breastfeeding duration and an objective measure of birth weight were recorded at birth. Children are significantly and increasingly less likely to be breastfed as birth order increases (P<. 0.00) [42]. Breastfeeding for six months or less has also been found to be an independent risk factor for mental health problems throughout childhood and adolescence (OR 1.33; 95% CI 1.09–1.62) [43]. There is a potential mechanism between higher birth order children, who are less likely to be breastfed, subsequently impacting mental health. Secondly, birth order is associated with birthweight—first order births are smaller than later births for men (3338g vs 3491, P<0.01) and women (3243g vs 3359g, P <0.01) [26]. Low birthweight is also associated to psychiatric illness in adulthood (OR 1.93; 95% CI (1.55–1.70) [44]. Thus, birth order may impact birthweight which can have a resultant impact on adult mental health.

Being of birth order one and having no subsequent siblings increases the risk of family dissolution (P < 0.01) [45, 46]. Psychiatric illnesses are also more common amongst those with separated parents (P < 0.01) [46]. Finally, first birth order has been found to significantly predict the risk of having an emotional disorder in childhood (P< 0.00) [46]. Childhood behaviour and conduct is also a risk factor for later life mental health problems (OR 1.7; 95% CI 1.03–2.70) [47], thus low birth order children, may be at an increased risk of emotional disorder, which subsequently may impact later life mental health.

Birth characteristics refer to exposures directly associated to the parents of the participants, must have occurred prior to the participant’s birth and were measured in the birth surveys. Birth characteristics accounted for included—maternal age, maternal smoking, parental education, mother’s marital status, father’s employment, parental social class and region of birth. Social class is strongly associated to negative health outcomes, larger family formations and increased sibship size [48]. Maternal smoking and maternal age, can detrimentally impacts offspring cognition, emotional and psychological wellbeing [49, 50] Parental relationship instability is associated to poor parental and childhood wellbeing [51–53]. Whilst, maternal age and parental marital status influences the opportunity to achieve larger completed sibship size. Parental education is also related to income, class and offspring education. Thus, lower educated parents can detrimentally impact offspring education, development and cognitive ability [54]. Finally, the prevalence of mental illnesses, level health, unemployment, social class, education and economic activity varies by region[55]. Additionally, this paper adjusted for several confounders in adulthood shown to be associated with mental wellbeing and psychological distress, including years of education, partnership formation and employment status as recorded at age forty-two.

### Analytical strategy

The conceptual diagram included in S1 Fig, illustrates the associations between birth order and mental wellbeing: i) Birth order directly effects the conditions an individual grows up which in turn impact mental wellbeing and psychological distress in midlife, ii) The relationships between birth order and mental wellbeing and psychological distress are mediated by birthweight, breastfeeding duration, parental separation and childhood conduct disorder; iii) The relationships between birth order and mental wellbeing and psychological distress are confounded by smoking during pregnancy, maternal age, mother’s legal marital status at birth, father’s employment at birth, region of birth, parental years of education and parental social class at birth and years of education, partnership formation and employment status in adulthood.

### Statistical methods

Firstly, descriptive statistics were conducted on the outcome variables. Logistic regression was carried out on the Malaise Index and the doctor’s consultation variable as binary outcomes, whilst. linear regression was used to analyse the WEMWBS. Model 1 tested the unadjusted association between birth order, mental wellbeing, psychological distress and doctor’s consultation. Model 2 tested the association between birth order and the three outcomes adjusting for birth characteristics (maternal age, mother’s legal marital status at birth, father’s employment at birth, region of birth, parental years of education and parental social class). Model 3 added mediators measured at birth to model 2 (birthweight and level of breastfeeding). Model 4 added mediators measured at age ten to model 3 (parental separation and conduct disorder). Model 5 additionally included confounders at age forty-two (partnership status, years of education and employment status). The associations were analysed independently for men and women.

## Results

Tables 1 and 2 presents the percentage at a high risk of malaise, the percentage who had attended a doctors consultation for a mental health issue and the distribution of WEMWBS scores according to birth order and childhood mediators analysed independently for men and women. There was no association between birth order and high risk of psychological distress or attending a doctor’s consultation. However, a larger portion of men (26.8%) who had severe behavioural issues at age ten were at high risk of psychological distress, compared to the portion of men with normal behaviour (17.4%). This difference was found to be statistically significant when regressed independently to the Malaise Index (1.40 95% CI 1.06, 1.86). Table 2 indicates that WEMWBS scores were on average slightly lower for men (48.59) and women (49.11) of birth order four and above, compared to birth order one (men 49.40; women 49.20). WEMWBS scores were lower for men (47.56) and women (48.81) with a severe behavioural issue at age 10.

**Table 1:** Risk of malaise and whether consulted doctor about a mental health issue: Bivariate associations with birth order and childhood mediators.

Variable		Malaise Index				Doctor’s Consultation			
		High Risk of Psychological Distress (score of 4–9)				Had a doctor’s consultation in the past year for a mental health issue			
		Men		Women		Men		Women	
		Percentage	Total sample number	Percentage	Total sample number	Percentage	Total sample number	Percentage	Total sample number
Birth Order	1	17.4%	1,641	19.3%	1,577	13.4%	1,876	13.5%	1,809
	2	17.4%	1,439	17.9%	1,329	12.7%	1,628	13.6%	1,532
	3	18.9%	699	21.9%	676	14.4%	807	15.0%	786
	4+	18.9%	647	17.2%	575	13.3%	750	13.3%	661
Birthweight	Low birthweight	18.8%	352	19.0%	341	12.0%	407	14.1%	396
	Normal birthweight	17.8%	4,042	19.0%	2,816	13.5%	4,617	13.7%	4,382
Level of Breastfeeding	Never breastfed	17.8%	2,801	18.8%	2,588	13.3%	3,203	13.3%	2,995
	Breastfed less than a month	17.9%	713	20.4%	672	13.4%	830	15.2%	764
	Breastfed more than a month	17.8%	914	18.5%	899	13.3%	1,030	13.9%	1,019
Parental Separation by age 10	Parents separated	17.6%	723	16.9%	651	12.3%	822	13.8%	744
	Parents not separated	17.7%	3,293	19.7%	3,109	13.6%	3,775	13.7%	3,585
Conduct Disorder at age 10	Normal behaviour	17.4%	2,392	18.4%	2,272	13.4%	2,731	13.5%	2,607
	Moderate behaviour	16.1%	466	20.6%	403	15.7%	517	15.5%	465
	Severe behaviour	26.8%	142	20.1%	149	9.8%	163	11.5%	166
	Incomplete information	18.7%	477	22.1%	429	12.4%	558	12.4%	491
	Single	21.0%	1,057	22.6%	1,019	16.0%	1,217	13.8%	1,181

**Table 2:** Mean and Standard Deviation of WEMWBS: Bivariate associations with birth order, and childhood mediators.

Variable		WEMWBS			
		Men		Women	
		Mean	Standard deviation	Mean	Standard deviation
Birth Order	1	49.40	8.26	49.20	8.32
	2	49.40	8.17	49.15	8.21
	3	48.91	8.42	49.06	8.85
	4+	48.59	8.51	49.11	8.14
Birthweight	Low birthweight	48.88	8.13	49.66	8.60
	Normal birthweight	49.23	8.31	49.10	8.32
Level of Breastfeeding	Never breastfed	49.20	8.35	49.16	8.35
	Breastfed less than a month	49.40	8.05	48.97	8.17
	Breastfed more than a month	49.05	8.34	49.27	8.46
Parental Separation by age 10	Parents separated	49.67	8.25	49.28	8.01
	Parents not separated	49.09	8.27	49.10	8.44
Conduct Disorder at age 10	Normal behaviour	49.35	7.83	49.19	8.21
	Moderate behaviour	49.01	9.23	49.25	8.95
	Severe behaviour	47.56	8.43	48.81	8.61
	Incomplete information	48.99	8.20	48.96	8.66

### Regression analysis

Model one in Tables 3 and 4 demonstrated no associations to the Malaise Index or doctor’s consultation. Model one in Table 5 indicated an unadjusted association between men of birth order four and above. Those men had a WEMWBS score 0.79 (95% CI;-1.57, -0.02) lower than first-born men, indicating a lower level of wellbeing at midlife. Model two indicated that when birth characteristics were accounted for this attenuated the significant association between birth order four plus and the WEMWBS, for men. Model three presented in Tables 3–5 tested the association mediated by birthweight and breastfeeding, the model provided no evidence for an association between birth order and the WEMWBS, the Malaise Index or doctor’s consultation for men or women.

**Table 3:** Odds ratios from logistic regression models testing the association between birth order and attending a doctor’s consultation in the past year for a mental health issue. Significant values are included in bold.

			Model 1	Model 2^2^	Model 3^3^	Model 4^4^	Model 5^5^
Men	**Birth Order**	1	REF	REF	REF	REF	REF
		2	1.06 (0.87, 1.29)	1.04 (0.84, 1.28)	1.05 (0.85, 1.29)	1.11 (0.86, 1.42)	1.12 (0.87, 1.44)
		3	0.92 (0.73, 1.17)	0.88 (0.68, 1.14)	0.89 (0.68, 1.15)	0.86 (0.63, 1.16)	0.85 (0.63, 1.16)
		4+	1.00 (0.78, 1.29)	0.97 (0.72, 1.30)	0.97 (0.72, 1.31)	0.87 (0.62, 1.22)	0.88 (0.62, 1.24)
	**Constant**		6.47	5.97	7.24	11.16	10.014
	**Sample number**		5,061	5,037	5,037	3,574	3,155
Women	**Birth Order**	1	REF	REF	REF	REF	REF
		2	1.00 (0.82, 1.22)	1.02 (0.83, 1.26)	1.02 (0.83, 1.27)	0.93 (0.72, 1.20)	0.94 (0.73, 1.21)
		3	0.89 (0.70, 1.13)	0.92 (0.71, 1.19)	0.92 (0.71, 1.19)	0.84 (0.61, 1.15)	0.84 (0.61, 1.15)
		4+	1.02 (0.79, 1.33)	1.09 (0.80, 1.48)	1.09 (0.80, 1.49)	0.91 (0.63, 1.31)	0.90 (0.63, 1.30)
	**Constant**		6.38	8.23	8.93	15.80	11.26
	**Sample number**		4,778	4,749	4,749	3,360	3,360

^1^ Model 1 unadjusted association.

^2^ Model 2 adjusted for smoking during pregnancy, maternal age, mother’s legal marital status at birth, father’s employment at birth, region of birth, parental years of education and parental social class.

^3^ Model 3 adjusted for smoking during pregnancy, maternal age, mother’s legal marital status at birth, father’s employment at birth, region of birth, parental years of education and parental social class. Mediators at birth (birth weight, level of breastfeeding).

^4^ Model 4 adjusted for smoking during pregnancy, maternal age, mother’s legal marital status at birth, father’s employment at birth, region of birth, parental years of education and parental social class. Mediators at birth (birth weight, level of breastfeeding) and mediators at age ten (parental separation, conduct disorder).

^5^ Model 5 adjusted for smoking during pregnancy, maternal age, mother’s legal marital status at birth, father’s employment at birth, region of birth, parental years of education and parental social class. Mediators at birth (birth weight, level of breastfeeding), mediators at age ten (parental separation, conduct disorder) and confounders in adulthood (years of education, employment status, partnership status).

**Table 4:** Odds ratios from logistic regression models testing the association between birth order and the Malaise Index. Significant values are included in bold.

			Model 1^1^	Model 2^2^	Model 3^3^	Model 4^4^	Model 5^5^
Men	**Birth Order**	1	REF	REF	REF	REF	REF
		2	1.00 (0.83, 1.21)	0.99 (0.81, 1.22)	0.98 (0.80, 1.19)	0.89 (0.70, 1.13)	0.87 (0.69, 1.11)
		3	1.11 (0.88, 1.39)	1.12 (0.87, 1.43)	1.08 (0.84, 1.39)	0.93 (0.69, 1.26)	0.93 (0.68, 1.26)
		4+	1.11 (0.88, 1.40)	1.11 (0.84, 1.47)	1.09 (0.83, 1.44)	1.11 (0.80, 1.54)	1.10 (0.79, 1.53)
	**Constant**		0.21	0.14	0.19	0.18	0.37
	**Sample number**		4,426	4,406	4,370	3,121	3,121
Women	**Birth Order**	1	REF	REF	REF	REF	REF
		2	0.91 (0.76, 1.10)	0.86 (0.70, 1.05)	0.85 (0.70, 1.04)	0.87 (0.69, 1.10)	0.91 (0.72, 1.16)
		3	1.17 (0.94, 1.46)	1.04 (0.81, 1.33)	1.02 (0.80, 1.31)	1.17 (0.88, 1.56)	1.15 (0.86, 1.54)
		4+	0.87 (0.68, 1.12)	0.76 (0.57, 1.02)	0.74 (0.55, 1.05)	0.75 (0.53, 1.06)	0.75 (0.53, 1.07)
	**Constant**		0.24	0.23	0.19	0.13	0.24
	**Sample number**		4.157	4,139	4,134	2,926	2,926

^1^ Model 1 unadjusted association.

^2^ Model 2 adjusted for smoking during pregnancy, maternal age, mother’s legal marital status at birth, father’s employment at birth, region of birth, parental years of education and parental social class.

^3^ Model 3 adjusted for smoking during pregnancy, maternal age, mother’s legal marital status at birth, father’s employment at birth, region of birth, parental years of education and parental social class. Mediators at birth (birth weight, level of breastfeeding).

^4^ Model 4 adjusted for smoking during pregnancy, maternal age, mother’s legal marital status at birth, father’s employment at birth, region of birth, parental years of education and parental social class. Mediators at birth (birth weight, level of breastfeeding) and mediators at age ten (parental separation, conduct disorder).

^5^ Model 5 adjusted for smoking during pregnancy, maternal age, mother’s legal marital status at birth, father’s employment at birth, region of birth, parental years of education and parental social class. Mediators at birth (birth weight, level of breastfeeding), mediators at age ten (parental separation, conduct disorder) and confounders in adulthood (years of education, employment status, partnership status).

**Table 5:** Linear regression coefficients from models testing the association between birth order and the WEMWBS. Significant values are included in bold.

			Model 1^1^	Model 2^2^	Model 3^3^	Model 4^4^	Model 5^5^
Men	**Birth Order**	1	REF	REF	REF	REF	REF
		2	0.00 (-0.61, 0.61)	0.08 (-0.58, 0.73)	0.09 (-0.57, 0.74)	-0.04 (-0.81, 0.73)	0.09 (-0.66, 0.83)
		3	-0.51 (-1.26, 0.24)	-0.50 (-1.33, 0.32)	-0.51 (-1.33, 0.32)	-0.31 (-1.29, 0.66)	-0.30 (-1.25, 0.65)
		4+	**-0.79 (-1.57, -0.02)**	-0.90 (-1.82, 0.02)	-0.86 (-1.78, 0.07)	**-1.19 (-2.28, -0.09)**	-1.04 (-2.11, 0.03)
	**Constant**		49.40	49.59	48.44	49.27	44.93
	**Sample number**		4,176	4,157	4,122	2,941	2,948
Women	**Birth Order**	1	REF	REF	REF	REF	REF
		2	-0.53 (-0.68, 0.58)	-0.11 (-0.78, 0.57)	-0.05 (-0.72, 0.62)	-0.21 (-0.99, 0.58)	-0.24 (-1.00, 0.52)
		3	-0.15 (-0.92, 0.63)	-0.17 (-1.03, 0.69)	-0.08 (-0.93, 0.77)	-0.45 (-1.45, 0.55)	-0.21 (-1.18, 0.77)
		4+	-0.10 (-0.92, 0.73)	-0.04 (-1.02, 0.94)	0.06 (-0.92, 1.04)	0.10 (-1.04, 1.25)	0.19 (-0.93, 1.30)
	**Constant**		49.21	47.19	48.53	48.57	43.82
	**Sample number**		3,899	3,883	3,878	2,750	2,750

^1^ Model 1 unadjusted association.

^2^ Model 2 adjusted for smoking during pregnancy, maternal age, mother’s legal marital status at birth, father’s employment at birth, region of birth, parental years of education and parental social class.

^3^ Model 3 adjusted for smoking during pregnancy, maternal age, mother’s legal marital status at birth, father’s employment at birth, region of birth, parental years of education and parental social class. Mediators at birth (birth weight, level of breastfeeding).

^4^ Model 4 adjusted for smoking during pregnancy, maternal age, mother’s legal marital status at birth, father’s employment at birth, region of birth, parental years of education and parental social class. Mediators at birth (birth weight, level of breastfeeding) and mediators at age ten (parental separation, conduct disorder).

^5^ Model 5 adjusted for smoking during pregnancy, maternal age, mother’s legal marital status at birth, father’s employment at birth, region of birth, parental years of education and parental social class. Mediators at birth (birth weight, level of breastfeeding), mediators at age ten (parental separation, conduct disorder) and confounders in adulthood (years of education, employment status, partnership status

Model four in Tables 3–5 illustrated the association mediated by conduct disorder and parental separation. Model four in Table 5 indicated that men of birth order four and above were associated with the WEMWBS. Those men had a WEMWBS score 1.19 (-2.28, -0.09) lower than first-born men, indicating a lower level of wellbeing at midlife. When childhood mediators were added to the model conduct disorder was found to attenuated this association. Tables 3 and 4 indicated that for model four there were no further associations between birth order and the Malaise Index or doctor’s consultation, for men or women.

Model five presented the fully adjusted model, showing the association between birth order, mental wellbeing and psychological distress, adjusting for birth characteristics and mediators at birth, age ten and confounders in adulthood. We find no evidence for a significant association between birth order and the WEMWBS, the Malaise Index or doctor’s consultation, for men or women. For men, the association between birth order four plus and the WEMWBS previously seen in model one and model four were attenuated when adulthood confounders were adjusted for (-1.04 CI -2.11, 0.03).

## Discussion

This study found no evidence to support the hypothesis that birth order was associated with mental wellbeing and psychological distress in midlife after considering confounding and mediating factors. There was little evidence to indicate differences in effect estimates when stratified by gender. Including conduct disorder at age ten attenuated the relationship between birth order and mental wellbeing at age 42. Adulthood socioeconomic confounders also attenuated any significant association between birth order and midlife outcomes.

The results did not support the resource depletion hypothesis. Investing increased resources into the surviving eldest child might have been a ‘Darwinist best bet’ in an historical era when childhood mortality was high and sibship sizes were large. However, the BCS70 were born in an era where childhood mortality was low and fertility dropped dramatically, while resources may have been more evenly distributed amongst siblings. Further, this study did not support previous findings including Barclay & Kolk study of 509,984 people in Sweden who indicated a relative gender effect of birth order on mortality that was greater among women (OR 1.46; 95% CI 1.27–1.67) than among men (OR 1.21; 95% CI 1.09–1.35) [29]. One explanation is that within the UK there no longer remains a preference for boys as such resources are now more likely to be equally distributed amongst genders.

This analysis found that socioeconomic confounders in adulthood including employment, partnership status and educational attainment were strongly associated with mental wellbeing and psychological distress at midlife, however early life factors were weakly associated. A potential explanation is that the long lead time between factors at birth, at age ten and the outcomes has resulted in any potential effect becoming diminished overtime. A second hypothesis, is that there may be other childhood mediators, unexplored within this study that are more important for predicting mental wellbeing. The finding that conduct disorder is associated with mental wellbeing in midlife postulates the conclusion that further research should focus on the development of personality types and behaviour across childhood.

In contrast to our findings, earlier work found associations between birth order and physical health in adulthood including, hospitalisation (P<0.01) amongst a sample of 10,394 British children [19] and mortality amongst a study of 407 US participants (P<0.01) [29]. Further, this study did not support earlier research that found an associated between first birth order, low birth weight and schizophrenia [13, 16, 22]. A potential explanation for this is that schizophrenia is a mental illness, whilst our research has utilised self-reported measure of psychological distress and mental wellbeing. The inconsistencies in the current evidence may be a result of differences either in the definition of birth order or the measurement of wellbeing and psychological distress utilised. There was also a wide variation in methodological approaches amongst studies; many utilised a cross-sectional or retrospective approach, analysing hospital records. The differences in association indicated may also have be dependent on the data analysed, the national context, and the age at which wellbeing was measured.

Our findings were similar to other studies that has found no evidence to support an association between birth order and other health outcomes in adulthood including both alcoholism [31] and anorexia in the US [25]. Further, our findings were consistent with another study based on the BCS70 [7]. The study concluded that early life factors including birthweight (men -0.01; 95% CI; -0.20–0.18; women -0.01; 95% CI; -0.27–0.04), maternal smoking (men 0.07; 95% CI; -0.02–0.17; women 0.16; 95% CI; 0.07–0.23) and breastfeeding (men -0.09; 95% CI -0.18–0.00; women -0.06; 95% CI -0.14–0.01) were modest at best when predicting psychological distress assessed via the Malaise Index in adulthood, although the paper did not specifically analyse birth order [7].

### Strengths and Limitations

There are several strengths of this analysis. These include the large sample size of the study, utilising one of the four major British birth cohorts, which is representative of the UK national population, although attrition may render the sample less representative than the initial birth sweep. Previous research into the BCS70 has found that attrition is greater for the mobile, disadvantaged men, participants whose fathers had a manual occupation or mothers with lower levels of education [38]. This were the same conclusion drawn from the drop out analysis conducted within this study and included in S1 and S2 Tables. As such, a limitation is that these individuals may have become less representative within the sample. We also cannot exclude the fact that those with severe mental health issues may have been more likely to drop out of the study. The use of prospective longitudinal data has provided lifelong data, placing the sample in a particular historical era and provides the opportunity to analyse the time-sequenced collection of data [56]. Knowing the temporal sequencing offers valuable means for elucidating an understanding of the causal effect. The study design of the BCS70 is therefore unique in that it offers the opportunity to address questions about processes of development and change over time [56].

The analysis also used three independent and alternative measures of mental wellbeing and psychological distress—capturing a more accurate and complete understanding of mental wellbeing and psychological distress in midlife. However, these measures of mental wellbeing are limited due to their self-reported nature. The analysis has also been limited in being unable to tease out the separation effects between birth order and maternal age; two variables that are strongly correlated to one another. The study also failed to capture the consequence of changing family structures within its definition of birth order. Birth order may have included sibships formed from several partnerships, whilst the analysis did not account for length of birth intervals, nor distinguished the gender of the previous births.

In summary, this study indicated that birth order may not be associated to mental wellbeing or psychological distress at midlife, for men or women. With the exception of conduct disorder childhood mediators were only weakly associated with adult mental wellbeing and psychological distress. However, socioeconomic and partnership status in adulthood appeared to be significant in attenuating any association to mental wellbeing and psychological distress at midlife.

### Future research

We conclude that on the evidence of this study the development of mental wellbeing is not a predefined pathway stemming from birth order. Instead, mental wellbeing and psychological distress results from individualised exposures across the life course. There is also a genetic component linked to the mental wellbeing and psychological distress that was not explored within this study.

Future research should use refined population cohort study data, with improved attrition rates and substantial pre-birth health markers, including an assessment of parental mental health, to test the conclusions drawn from this study. Future research should also look to develop and incorporate more objective measures of psychological distress and mental wellbeing.

## Supporting information

S1 Table Loss to follow up: Sample characteristics at birth for all respondents at birth and respondents followed up at age 42. (PDF)

S2 Table Loss to follow up: Sample characteristics at 10 for respondents at age 10 and respondents followed up at age 42. (PDF)

S1 Fig Analytical framework. (PDF)
