# Supplementary material for: Associations between birth order with mental wellbeing and psychological distress in midlife: Findings from the 1970 British Cohort Study (BCS70)
Source: PLoS One. 2019 Sep 17;14(9):e0222184. doi: 10.1371/journal.pone.0222184 (PMC6748419; doi:10.1371/journal.pone.0222184)
Supplement: S1 Table — (PDF) [file pone.0222184.s001.pdf]

S1 Table Loss to follow up: sample characteristics at birth for all respondents at birth and respondents followed up at age 42.

Variables at birth		**Men (%)**		**Women (%)**	
		All participants at birth	*Participants at age 42*	*All participants at birth*	*Participants at age 42*
Birth Order	*1*	36.8	38.3	38.2	39.6
	2	32.6	34.4	31.7	32.8
	3	16.0	15.8	16.0	15.6
	4+	14.6	11.6	14.1	11.9
Maternal Smoking Status During Pregnancy	*Non-smoker*	53.5	56.9	53.4	55.5
	Stopped during pregnancy	4.6	4.9	4.9	4.9
	Smoking during pregnancy	41.9	38.2	41.7	39.6
Mother's Age of Delivery	*18 and under*	5.7	5.0	5.7	4.7
	19-24 years	39.3	39.5	39.2	39.6
	25-29 years	31.3	32.8	31.3	32.7
	30-34 years	15.2	14.8	15.1	14.9
	35+ years	8.4	8.0	8.8	8.1
Parental Marital Status	*Single*	5.5	3.5	5.9	4.3
	Married	92.5	94.9	92.3	94.3
	Previously Married	2.0	1.6	1.8	1.4
Birth Region	*North*	6.1	6.5	5.8	6.6
	Yorkshire & Humber	8.3	8.9	9.0	9.2
	East Midlands	6.0	6.8	6.1	6.4
	East Anglia	3.3	3.5	3.0	3.4
	South East	29.1	29.3	29.3	29.6
	South West	5.7	6.5	6.5	7.6
	West Midlands	10.3	11.0	10.0	10.4
	North West	12.7	13.3	12.5	12.9
	Wales	5.2	5.6	5.0	5.2
	Scotland	9.6	8.6	9.2	8.6
	Northern Ireland	3.6	0.7	3.7	0.2
Parental Social Class	*Unskilled/ partly skilled*	23.4	19.4	23.3	21.2
	Manual	44.6	44.7	44.2	44.0
	Non-manual	13.6	15.1	13.4	14.1
	Managerial/Professional	18.0	20.5	18.2	20.3
	Other or Not stated	0.5	0.3	0.8	0.5
Father's Employment	*Employed*	88.5	91.7	87.8	90.1
	Not employed	3.5	2.5	3.6	2.7
	Not stated/No Father	8.0	5.8	8.6	7.2
Breastfeeding	*No Breastfeeding*	63.1	63.1	63.4	63.8
	Less that 1 month	16.3	16.4	16.0	15.7
	More than 1 month	20.6	20.5	20.6	20.4
Birthweight	*Normal birthweight*	92.5	94.7	91.7	93.7
	Low birthweight	7.5	5.3	8.3	6.3
Total sample size		8,463	4,346	8,290	4,746
