# Supplementary material for: Associations between birth order with mental wellbeing and psychological distress in midlife: Findings from the 1970 British Cohort Study (BCS70)
Source: PLoS One. 2019 Sep 17;14(9):e0222184. doi: 10.1371/journal.pone.0222184 (PMC6748419; doi:10.1371/journal.pone.0222184)
Supplement: S2 Table — (PDF) [file pone.0222184.s002.pdf]

S2 Table Loss to follow up: sample characteristics at 10 for respondents at age 10 and respondents followed up at age 42.

Variables at age 10		**Men**		**Women**	
		Participants at age 10	*Participants at age 42*	*Participants at age 10*	*Participants at age 42*
Parental separation	*Parents separated*	13.4	12.2	14.2	13.6
	Parents not separated	86.6	87.8	85.8	86.4
Rutter Behavioural scale	*Normal behaviour*	66.5	70.5	69.2	74.9
	Moderate behaviour	14.7	14.2	12.6	11.3
	Severe behaviour	4.9	4.0	4.5	3.3
	Not stated	13.9	11.4	13.7	10.5
Total sample size		6,982	4,050	6,485	4,436
