# Supplementary figures and images for: Associations between birth order with mental wellbeing and psychological distress in midlife: Findings from the 1970 British Cohort Study (BCS70)
Source: PLoS One. 2019 Sep 17;14(9):e0222184. doi: 10.1371/journal.pone.0222184 (PMC6748419; doi:10.1371/journal.pone.0222184)

*S1 Fig Analytical framework.*

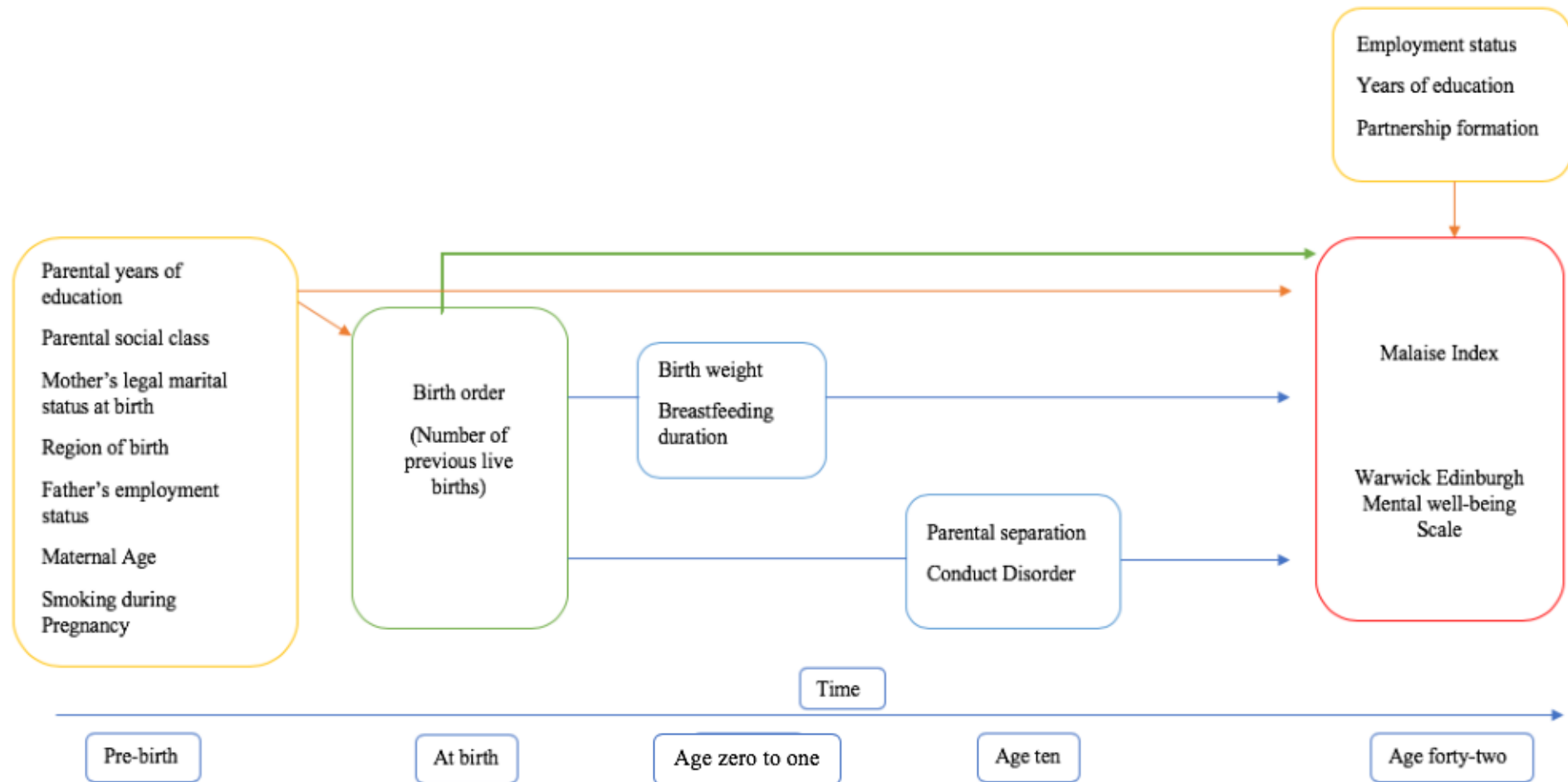

Supplement: S1 Fig — (PDF) [file pone.0222184.s003.pdf]
